# Supplementary material for: Combined Effect of Naturally-Derived Biofilm Inhibitors and Differentiated HL-60 Cells in the Prevention of Staphylococcus aureus Biofilm Formation
Source: Microorganisms. 2020 Nov 9;8(11):1757. doi: 10.3390/microorganisms8111757 (PMC7695255; doi:10.3390/microorganisms8111757)
Supplement: Supplementary file 1 [file microorganisms-08-01757-s001.pdf]

Table S1 previously reported antimicrobial activity and anti-biofilm potencies of the biofilm inhibitors. DHA1 and DHA2 are coded 11 and 9b, respectively in [1] while FLA1 is coded 291 in [2].

	Biofilms IC_{50} (μM)		Planktonic bacteria
	**Pre-exposure**	**Post-exposure**	**MIC (μM)**
**DHA1**	9.4	27.9	15
**DHA2**	33.2	86.1	60
**FLA1**	10.2	27.9	20

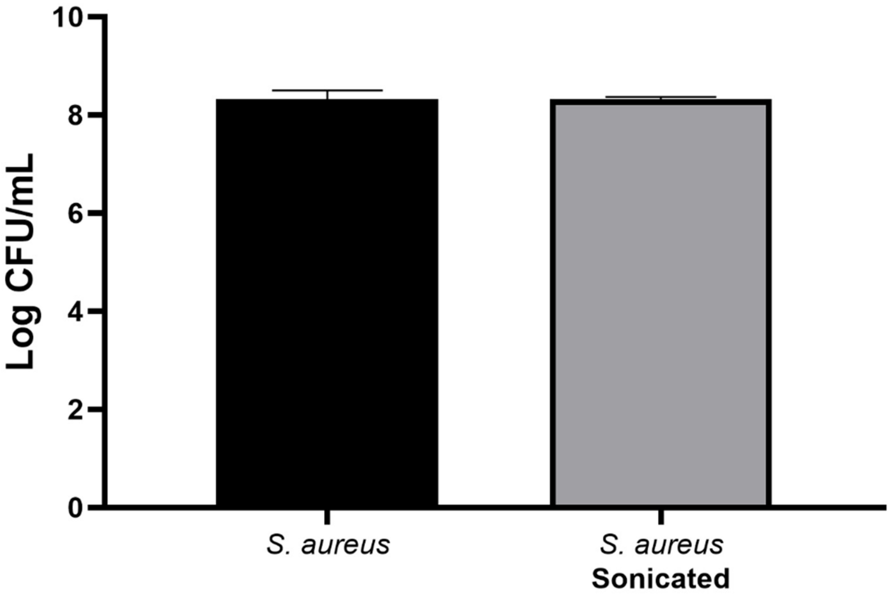

Figure S1 Comparison between viable *S. aureus* ATCC 25923 after exposing an inoculum of 10^8 CFU/mL to 10 min sonication at 35 kHz. Results expressed as mean + SD (n=3), experiment repeated twice.

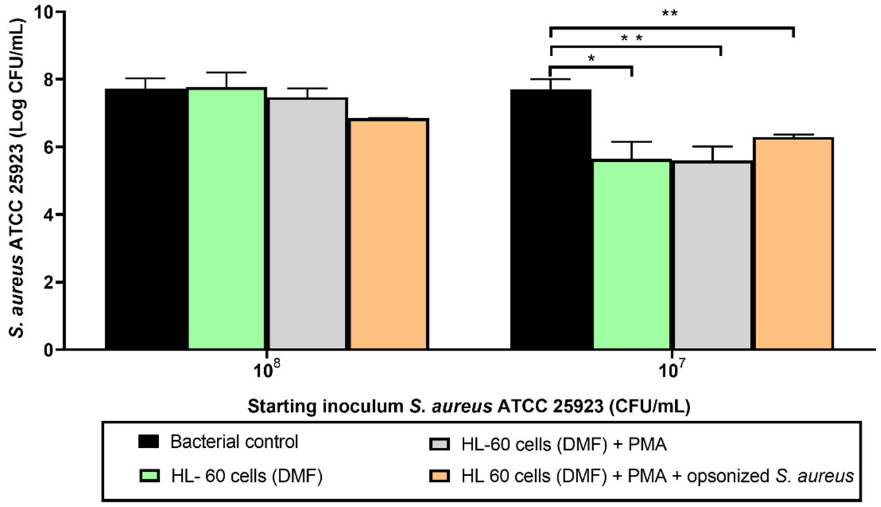

Figure S2 Viable counts of 24 h old biofilms formed by different concentrations of *S. aureus* ATCC 25923 co-cultured with HL-60 cells on 96-well microplates. Black columns represent the bacterial control (viable attached cells in the absence of HL-60 cells). Green columns show the co-culture of *S. aureus* ATCC 25923 with HL-60

cells differentiated with DMF. Grey columns show the co-culture of *S. aureus* ATCC 25923 with HL-60 cells differentiated with DMF and activated with PMA. Orange columns represent the co-culture of opsonized S. aureus with HL-60 cells differentiated with DMF and activated with PMA. Values are means and SD of two or more independent experiments (*** $p < 0.001$; ** $p < 0.01$; p* < 0.05)

References

1. Manner, S.; Vahermo, M.; Skogman, M.E.; Krogerus, S.; Vuorela, P.M.; Yli-Kauhaluoma, J.; Fallarero, A.; Moreira, V.M. New derivatives of dehydroabietic acid target planktonic and biofilm bacteria in *Staphylococcus aureus* and effectively disrupt bacterial membrane integrity. *European journal of medicinal chemistry* **2015**, *102*, 68-79, doi:10.1016/j.ejmech.2015.07.038.
2. Manner, S.; Skogman, M.; Goeres, D.; Vuorela, P.; Fallarero, A. Systematic Exploration of Natural and Synthetic Flavonoids for the Inhibition of *Staphylococcus aureus* Biofilms. *International journal of molecular sciences* **2013**, *14*, 19434-19451, doi:10.3390/ijms141019434.
